# **Robust immune response stimulated by in situ injection of CpG/**α**OX40/cGAMP in αPD-1-resistant malignancy**

**DOI:** 10.1007/s00262-021-03095-z

**Published:** 2021-11-03

**Authors:** Luya Cai, Xuedan Du, Cheng Zhang, Shanshan Yu, Lixiao Liu, Jinduo Zhao, Ye Zhao, Chunhong Zhang, Jinting Wu, Bin Wang, Yingyu Chen, Xiaoping Su, Xiaojian Yan, Wenfeng Li

**Affiliations:** 1 Department of Obstetrics and Gynecology, The First Affiliated Hospital of Wenzhou Medical University, Wenzhou, Zhejiang People’s Republic of China; 2 Department of Oncology, The First Affiliated Hospital of Wenzhou Medical University, Wenzhou, Zhejiang People’s Republic of China; 3 Department of Dermatology, Taizhou Hospital of Zhejiang Province, Linhai, Zhejiang China; 4 Department of Pharmacy, The First Affiliated Hospital of Wenzhou Medical University, Wenzhou, Zhejiang People’s Republic of China; 5 School of Basic Medical Science, Wenzhou Medical University, Wenzhou, Zhejiang People’s Republic of China; 6 Center for Uterine Cancer Diagnosis and Therapy Research of Zhejiang Province, Women’s Hospital and Institute of Translation Medicine, Zhejiang University School of Medicine, Hangzhou, China; 7 Department of Neurosurgery, The First Affiliated Hospital of Wenzhou Medical University, Wenzhou, Zhejiang People’s Republic of China

**Keywords:** CpG/αOX40/cGAMP, In situ vaccination, αPD-1-resistant malignancies, Tumour immune microenvironment

## Abstract

Recently, the emergence of immunotherapy has revolutionized traditional tumour treatment. However, effective treatments for patients exhibiting αPD-1 resistance are still lacking. In our study, a combination of cytosine–phosphate–guanine oligodeoxynucleotides (CpG-ODNs), anti-OX40 and cyclic guanosine monophosphate–adenosine monophosphate (cGAMP) injection in situ systematically generated a robust antitumour immune response in TC1 and B16 cells, which are αPD-1-resistant malignancies. More precisely, this method activates both adaptive and innate immunity. Additionally, in situ vaccination with CpG/αOX40/cGAMP fully activates the production of cytokines. However, the combination of αPD-1 does not improve the efficacy of triple therapy, prompting further questions. Collectively, the combination of CpG/αOX40/cGAMP causes the regression of various αPD-1-resistant tumours through the full mobilization of innate and adaptive immunity. In addition, we explored the therapeutic effect of triple therapy on the αPD-1-sensitive cell line CT26. The results showed that triple therapy could significantly enhance the therapeutic effect of αPD-1, and some mice even achieved complete tumour regression after the combined application of αPD-1 and triple treatment.

**Supplementary Information:**

The online version contains supplementary material available at 10.1007/s00262-021-03095-z.

## Introduction

The incidence and mortality rates of cancer are increasing gradually and are primary factors threatening human health. According to global cancer statistics, in 2018, approximately 18.1 million new cases of cancer were diagnosed worldwide, and approximately 9.6 million deaths occurred [1]. Currently, the traditional treatment methods for cancer include surgery, radiotherapy, chemotherapy and targeted therapy; however, each of these treatment methods has limitations, so the recurrence and metastasis of tumours remains a challenge, leading to a poor prognosis and low long-term survival rate in patients with advanced tumours. Therefore, studies exploring safer and more effective tumour treatment methods are urgently needed.

In recent years, the advent of immunotherapy has opened a novel chapter in the diversified treatment of various tumours, providing new hope for patients with cancer, particularly for some patients with advanced cancers. Currently, tumour immunotherapy is mainly divided into four categories according to the method: non-specific immune modulators, adoptive cell therapy, immune checkpoint inhibitors and tumour vaccines. Among them, the most common immunotherapy is PD-1/PD-L1 immune checkpoint inhibitors, which have been administered to many patients with cancer and have achieved a high response rate and lasting remission, significantly improving the survival rate of patients with recurrent or refractory Hodgkin's lymphoma [2–4], non-small cell lung cancer (NSCLC) [5–7], melanoma [8–10], etc., showing broad application prospects. Nevertheless, a large number of patients with cancer display a poor or ineffective response to PD-1/PD-L1 immune checkpoint inhibitors. Thus, an effective treatment must be developed for αPD-1-resistant patients.

In addition, in situ vaccination is a promising antitumour treatment method in which immune-enhancing agents are directly injected into the tumour site to stimulate the antitumour immune response throughout the body by inducing antigen release, promoting antigen presentation, activating immune effector cells, etc. [11]. A growing number of studies have focused on the antitumour efficacy of in situ vaccines. For example, Levy et al. found that the intratumour injection of low-dose cytosine–phosphate–guanine oligodeoxynucleotides (CpG-ODNs) induces the expression of OX40 on CD4^+^ T cells, and the combination of CpG with agonistic anti-OX40 antibody further enhances the antitumour effect of immune cells, systematically shrinking the tumours in mice, particularly lymphoma [12]. Temizoz et al. found that intratumour injections of CpG and cGAMP effectively inhibited tumour growth in EG-7 and B16 F10 mouse tumour models [13]. According to Monjazeb et al., systemic blockade of the immunosuppressive enzyme indolamine-2,3-dioxygenase (IDO) combined with CpG/radiotherapy in situ induces robust systemic antitumour effects [14]. Based on the excellent preclinical research results, a number of clinical trials were initiated. In patients with low-grade B-cell lymphoma or mycosis fungoides (MF), systemic clinical responses to the combination of low-dose radiotherapy and CpG treatment have been observed in one tumour without serious treatment-related adverse events (NCT00185965, NCT00226993) [15, 16]. In patients with indolent non-Hodgkin’s lymphomas (iNHLs), in situ vaccination with Flt3L, radiotherapy and a TLR3 agonist induced systemic clinical tumour regression (NCT01976585) [17]. However, researchers have not clearly determined whether in situ vaccines exert a therapeutic effect on tumours that do not respond to immune checkpoint inhibitors.

**Fig. 1:**
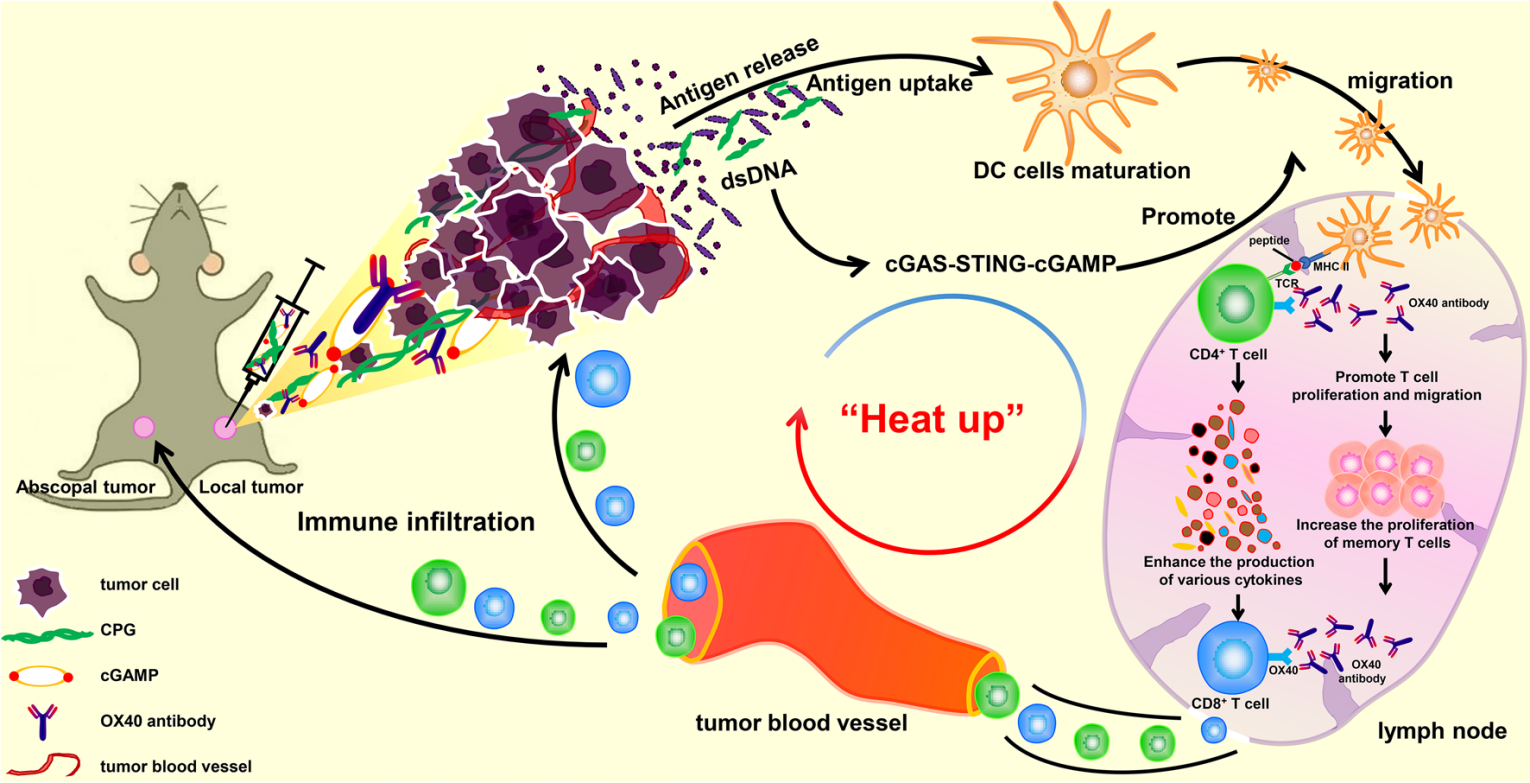
Schematic diagram of the therapeutic mechanism by which triple therapy induces systemic antitumour effects

An extensive preliminary study and screening were performed to identify an in situ vaccine with good efficacy against αPD-1-resistant tumours. Notably, the combination of cyclic guanosine monophosphate–adenosine monophosphate (cGAMP), a STING agonist and CpG/αOX40 exerted a powerful vaccine effect; specifically, systemic tumour growth was significantly slowed, and surprising results were observed. In addition, in situ vaccination with CpG/αOX40/cGAMP increased the infiltration of immune cells in distant tumours and throughout the body, fully mobilized the production of cytokines and simultaneously activated adaptive and innate immunity (Fig. 1). Moreover, when we combined αPD-1 with CpG/αOX40/cGAMP, the antitumour effect was not enhanced but was instead inhibited to some extent.

## Methods

### Mice

Female C57BL/6 mice and Balb/c mice (6–8 weeks old) were purchased from Hangzhou Ziyuan Experimental Animal Technology Co., Ltd. All mice were housed under specific pathogen-free conditions and maintained at a constant temperature (22 ± 1 °C) and humidity (60–70%). In addition, all animals were provided ultrapure water and clean food and housed on 12 h light/dark cycles. All animal experimental procedures were conducted in accordance with guidelines for the care and use of laboratory animals from the National Institutes of Health and were approved by the Animal Experimental Ethics Committee of Wenzhou Medical University.

### Cell lines and reagents

The TC1, B16 and CT26 cell lines were purchased from the Cell Bank of Chinese Academy of Science (Shanghai, China). They were cultured in RPMI 1640 supplemented with 10% foetal bovine serum (FBS) (Sigma), 100 μg/mL penicillin and 100 μg/mL streptomycin in a humidified incubator at 37 °C with 5% CO_2_.

CpG-ODN 2395 was provided by Synbio Technologies (Suzhou, China). The agonistic anti-OX40 antibody was provided by BioXcell (West Lebanon, USA). 2′3’-cGAMP was purchased from InvivoGen (Cat.# tlrl-nacga23m). CD4-, CD8- and NK-depleting antibodies were purchased from BioXcell (USA).

### Tumour inoculation and animal experiment

For the construction of a mouse model for the αPD-1 treatment experiment, the left flank of each C57BL/6 mouse was subcutaneously injected with 2 × 10^5^ TC1/B16 cells (50 μL of PBS) on day 0. When the tumour volume reached 10–20 mm^3^ on day 6 or 7, the mice were randomly divided into 2 groups: the PBS and αPD-1 groups. PBS or αPD-1 (100 μg/mouse) was administered by intraperitoneal injection every 2 days for 3 injections.

For the mouse model of systemic tumour, the left flank of each C57BL/6 mouse was subcutaneously injected with 2 × 10^5^ TC1/B16 cells (50 μL of PBS) (primary tumour) on day 0, and the same number of TC1/B16 cells was injected subcutaneously on the other side on day 2. On day 7, when the TC1 tumour volume reached 10–20 mm^3^, the mice were randomly divided into the following 5 groups: PBS, CpG, cGAMP, CpG/αOX40 and CpG/αOX40/cGAMP. CpG (50 μg/mouse), αOX40 (30 μg/mouse) and cGAMP (10 μg/mouse) were administered by in situ injection only into the primary tumour every 2 days for 3 injections. When the B16 tumour volume reached 5–10 mm^3^, the treatment began.

For the cell depletion experiment, anti-CD4 (clone GK1.5, BioXCell), anti-CD8 (clone 2.43, BioXCell) or anti-NK (clone PK136, BioXCell) mAbs were intraperitoneally injected at a dose of 200 μg/mouse one day before therapy. When the treatment began, each mouse was injected with 100 μg of the anti-CD4, anti-CD8 or anti-NK mAbs every 2 days for 5 injections. Finally, a flow cytometry analysis of blood samples was performed to validate whether CD4^+^ T cells, CD8^+^ T cells or NK cells were successfully knocked out. The tumour dimensions were measured with Vernier callipers every 2 or 3 days, and the tumour volume was calculated using the following equation: length × width^2^/2 (mm^3^). Mice were killed when the tumour volume reached 1500 mm^3^.

To explore the efficacy of the triple drug in the PD-1-sensitive cell line CT26, we subcutaneously injected 4 × 10^5^ CT26 cells into the left flank of Balb/C mice (primary tumour) and 2 days later injected the same number of CT26 cells into the right side (distant tumour). Mice were randomly divided into 4 groups—the PBS group, αPD-1 group, CpG/αOX40/cGAMP triple group and CpG/αOX40/cGAMP/αPD-1 quadruple group—and treated on day 5 with the same drug dose as previously described. The tumour size and body weight of the mice were observed every 2–3 days. The mice were killed when the tumour volume reached 1500 mm^3^.

### Flow cytometry analysis

Mouse spleen tissues were completely ground in the filter and digested with a tumour dissociation kit (Miltenyi Biotec Inc.) at 37 °C for 1 h, and the suspension was filtered through 40 μm sieves to obtain a single-cell suspension. Next, the single-cell suspension was placed in 0.02% saline for approximately 20 s and then neutralized with an equal volume of 0.16% saline to lyse the erythrocytes. One hundred microlitres of the single-cell suspension (~ 10^6^ cells) was stained with CD45-APC-A750, CD3e-FITC, CD4-APC, CD8-PC7, CD3-APC, CD11c-FITC and MHC II-PE antibodies and incubated at 4 °C in the dark for 30 min. After washing with staining buffer, a CyFLEX flow cytometer (Beckman Coulter) was used to conduct flow cytometry. The voltage was regulated by a negative control and single dye tube. The final data were analysed using CytExpert software.

### Cytokine assay

Blood samples were collected from blood vessels into a tube without anticoagulant and centrifuged twice for 20 min (4 °C, 3000 G) after incubation at room temperature for 2 h, and the supernatant was collected to analyse the changes in cytokine levels in the treated mice. The serum levels of Th1 (IL-2, IFN-γ and TNF), Th2 (IL-4, IL-6 and IL-10) and Th17 (IL-17A) cytokines were detected using a CBA kit according to the manufacturer’s instructions.

### Statistical analysis

All statistical analyses were performed using GraphPad Prism 7.0 software and SPSS 16.0. All data are presented as means ± SEM. Two-way ANOVA was used to examine the differences between groups, and *P* < 0.05 was considered significant.

## Results

### αPD-1 treatment did not produce a therapeutic benefit in mouse models of cervical cancer and melanoma

**Fig. 2:**
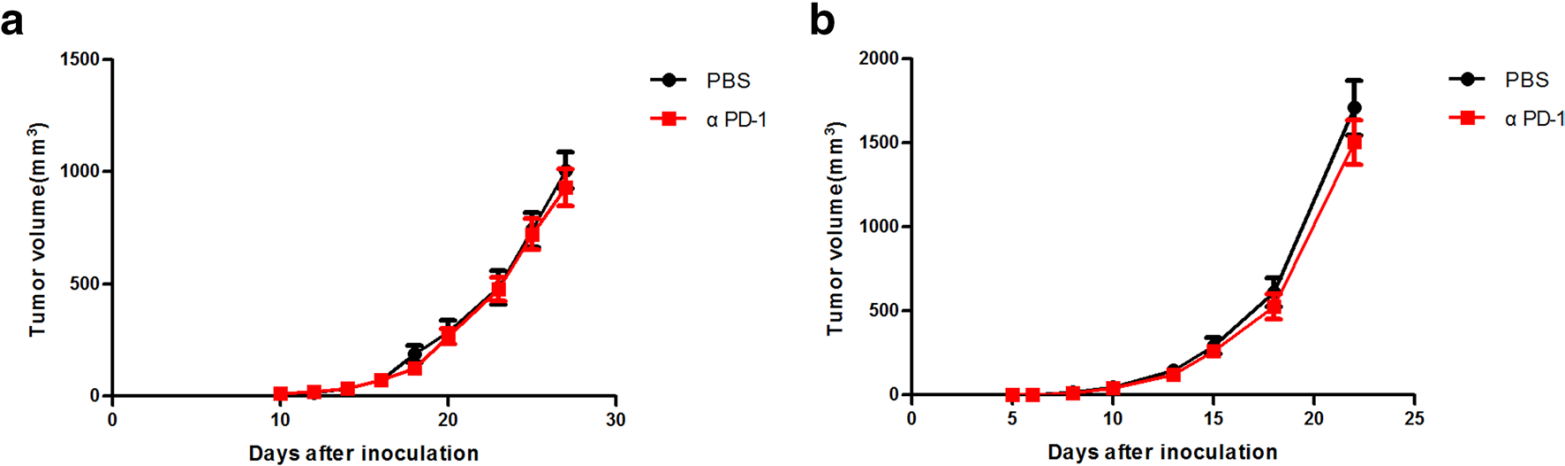
TC1/B16 tumours do not respond to αPD-1 immune checkpoint inhibitors. **a** TC1 and **b** B16

Mice bearing TC1/B16 tumours were treated with αPD-1 (100 μg/mouse) 3 times, but no difference in tumour volume was observed between the treatment group and the PBS group (Fig. 2a–b). Therefore, we concluded that these two types of cells are indeed αPD-1-resistant, consistent with previous studies [18–20]. We chose TC1/B16 cell lines for follow-up studies.

### CpG/αOX40/cGAMP exerted a systemic antitumour effect on αPD-1-resistant tumours

**Fig. 3:**
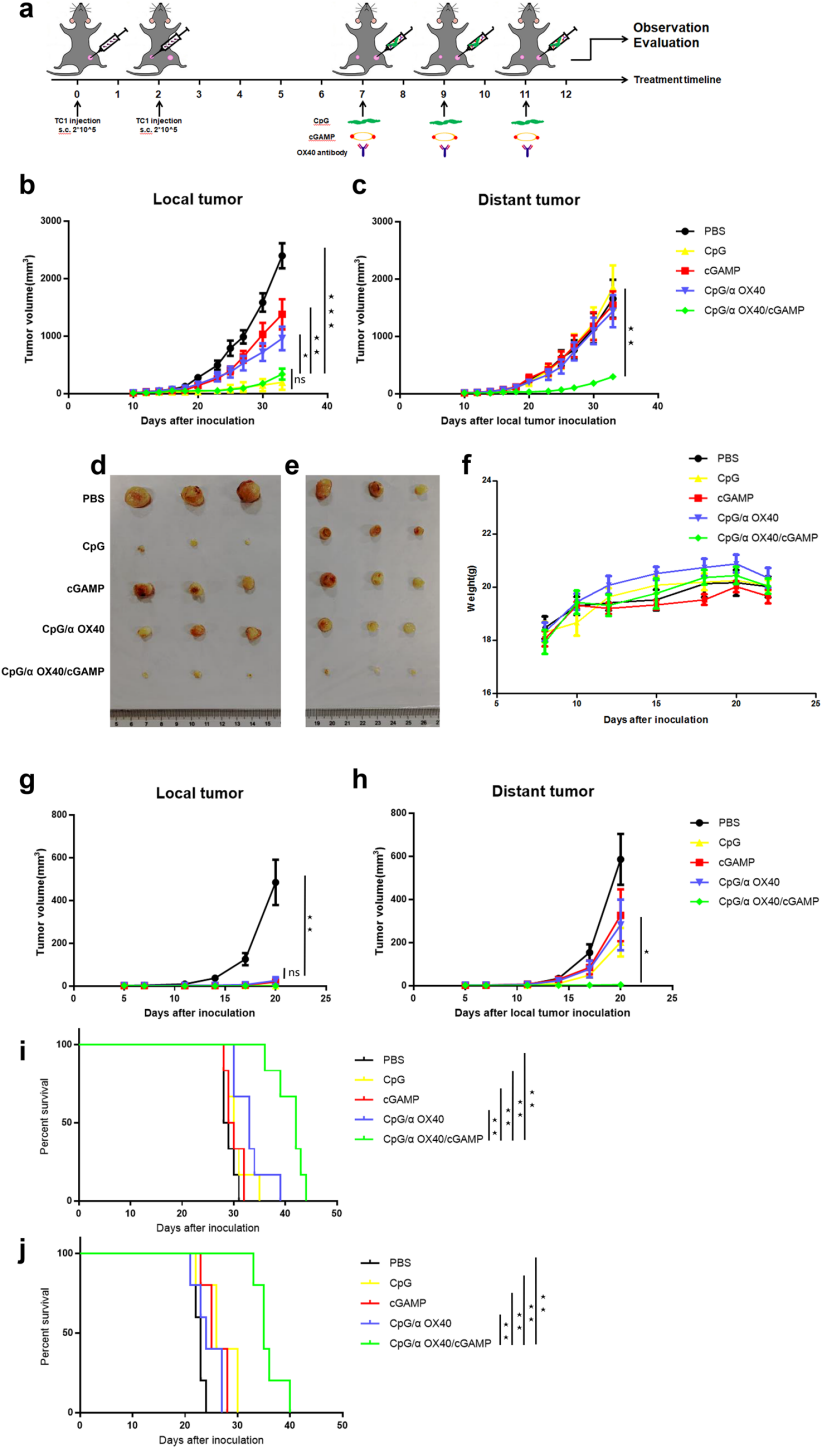
CpG/αOX40/cGAMP therapy exerted a systemic antitumour effect on αPD-1-resistant tumours. **a** Treatment flowchart for TC1 mouse models. C57BL/6 mice were subcutaneously injected with 2 × 10^5^ TC1 cells (50 µL of PBS) in the left flank (local tumour) on day 0 and in the right flank (distant tumour) on day 2. The treatment began on approximately day 7, when the tumour volume reached 10–20 mm^3^. The TC1 tumour growth curves for the treated side (**b**) and untreated side (**c**) are shown. Pictures of TC1 tumours harvested from the treated side (**d**) and untreated side **e** of mice are shown. **f** Changes in the weight of TC1 tumour-bearing mice. B16 tumour growth curves for the treated side (**g**) and untreated side (**h**) are shown. CpG/αOX40/cGAMP therapy significantly prolonged the survival time of TC1 (**i**) and B16 (**j**) tumour-bearing mice

A mouse model of αPD-1-resistant tumours was established, and all the treatments were only administered to the primary tumour to study the systemic efficacy of an in situ vaccine in αPD-1-resistant tumours (Fig. 3a). For mice bearing TC1 tumours, the growth rate of local tumours in the CpG, cGAMP, CpG/αOX40 and CpG/αOX40/cGAMP groups was significantly slower than that in the PBS group, particularly in the triple therapy group (Fig. 3b). However, when we observed distant tumours, the tumour growth rate of the other treatment groups was not slower, with the exception of the triple therapy group (Fig. 3c). In addition, we more intuitively observed an obvious difference in the tumour volume in each group from the photographs of tumours in mice (Fig. 3d–e). We concluded that the administration of this type of triple therapy to TC1 tumours significantly inhibited tumour growth and produced a systemic antitumour effect that was significantly stronger than the single-drug or CpG/αOX40 therapy. In addition, no significant difference in body weight was detected among these five groups, and no weight loss occurred after treatment, indicating that triple therapy was safe (Fig. 3f). Similar results were obtained for mice bearing B16 tumours (Fig. 3g–h), and triple therapy was the most effective.

Furthermore, we evaluated the survival time of tumour-bearing mice. The average survival time of TC1 tumour-bearing mice in the control group, namely the PBS group, was less than 30 days, while the survival time of mice treated with triple therapy was significantly prolonged to more than 40 days, with *P* < 0.01. The survival time of mice in the other treatment groups was not significantly different from that of the PBS group (Fig. 3i). Triple therapy also significantly prolonged the survival time of B16 tumour-bearing mice (Fig. 3j).

### The efficacy of triple therapy in modulating adaptive immunity in mice

**Fig. 4:**
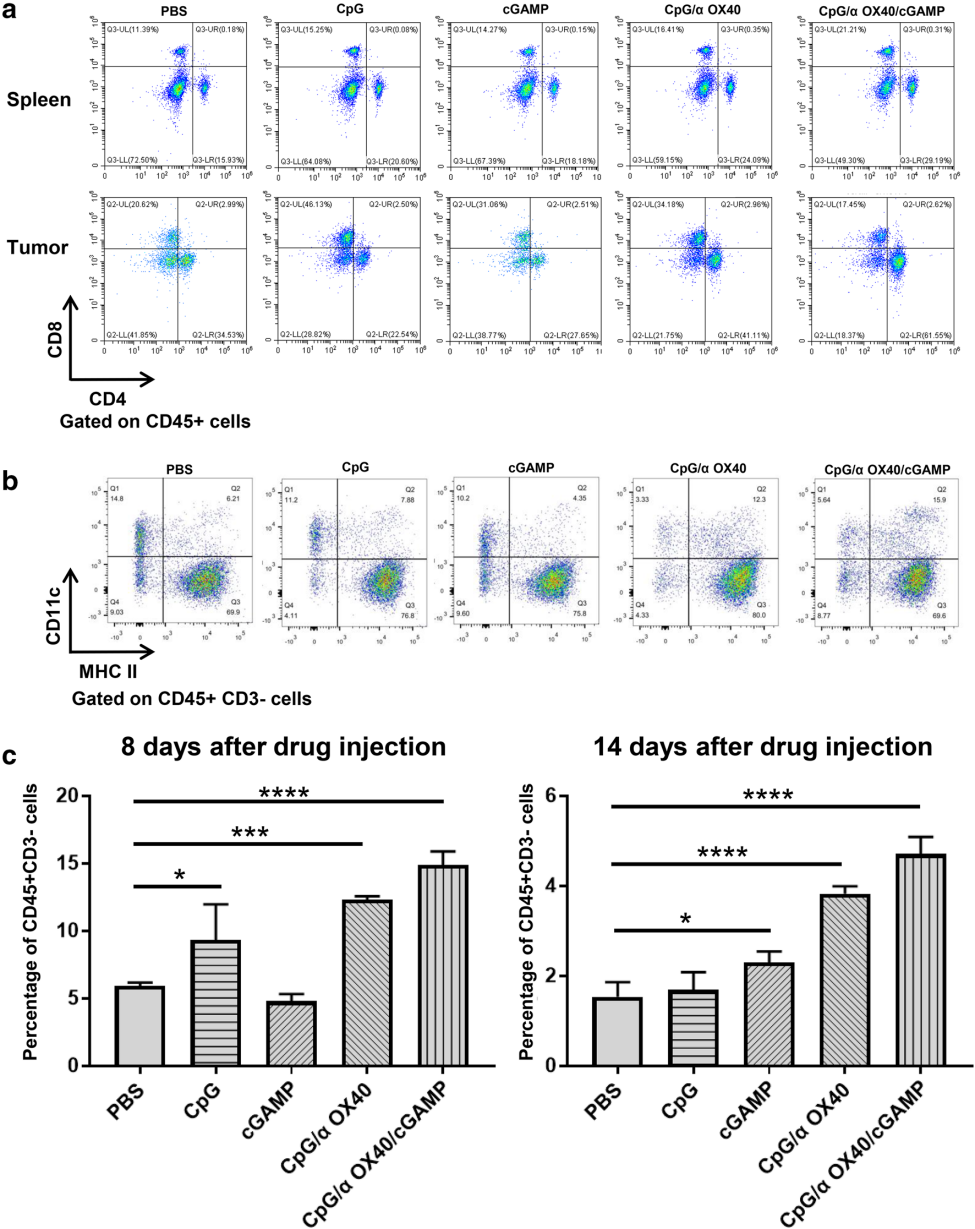
The efficacy of triple therapy depends on adaptive immunity in mice. **a** Proportions of CD4^+^ and CD8^+^ T cells among CD45^+^ cells in the mouse spleen and distant tumour. **b** Proportions of DCs in the mouse spleen on the 8th day after treatment. **c** Comparison of DC content among groups on the 8th (left) and 14th (right) days after treatment (the bar graphs represent the quantitative analysis of three independent experiments)

We analysed the changes in T cells and DCs in mice to explore the effect of triple therapy on adaptive immunity. Using flow cytometry, we found that the proportions of CD3^+^, CD4^+^ and CD8^+^ T cells in the spleens of the triple therapy group of mice were noticeably increased, and the proportion of T cells was approximately twice as high as that in the PBS group (Fig. 4a). In the other treatment groups, although T cells were also increased to varying degrees, the changes were not as obvious as in the triple therapy group (Fig. 4a). Regarding the intratumoural T cells in the untreated tumours, interesting phenomena were observed. When the mice were treated with CpG/αOX40 therapy, the proportions of both CD4^+^ and CD8^+^ T cells were remarkably increased compared to those in the PBS, CpG and cGAMP groups, which was consistent with previous studies. However, in the CpG/αOX40/cGAMP group, the proportion of CD3^+^ T cells was also strikingly increased and was the highest among all treatment groups, but the proportion of CD8^+^ T cells showed a slight decreasing trend, and the proportion of CD4^+^ T cells was substantially increased from 25 to 60% (Fig. 4a). In addition, we also detected changes in DC cells in the spleens of mice. From the results, we can see that although CpG alone can induce the maturation of DCs, its effect was not ideal and lasting. When combined with αOX40 and cGAMP, the proliferation of DCs was the most significant (Fig. 4b–c).

### The antitumour effect of triple therapy depends on the full mobilization of adaptive immunity and innate immunity

**Fig. 5:**
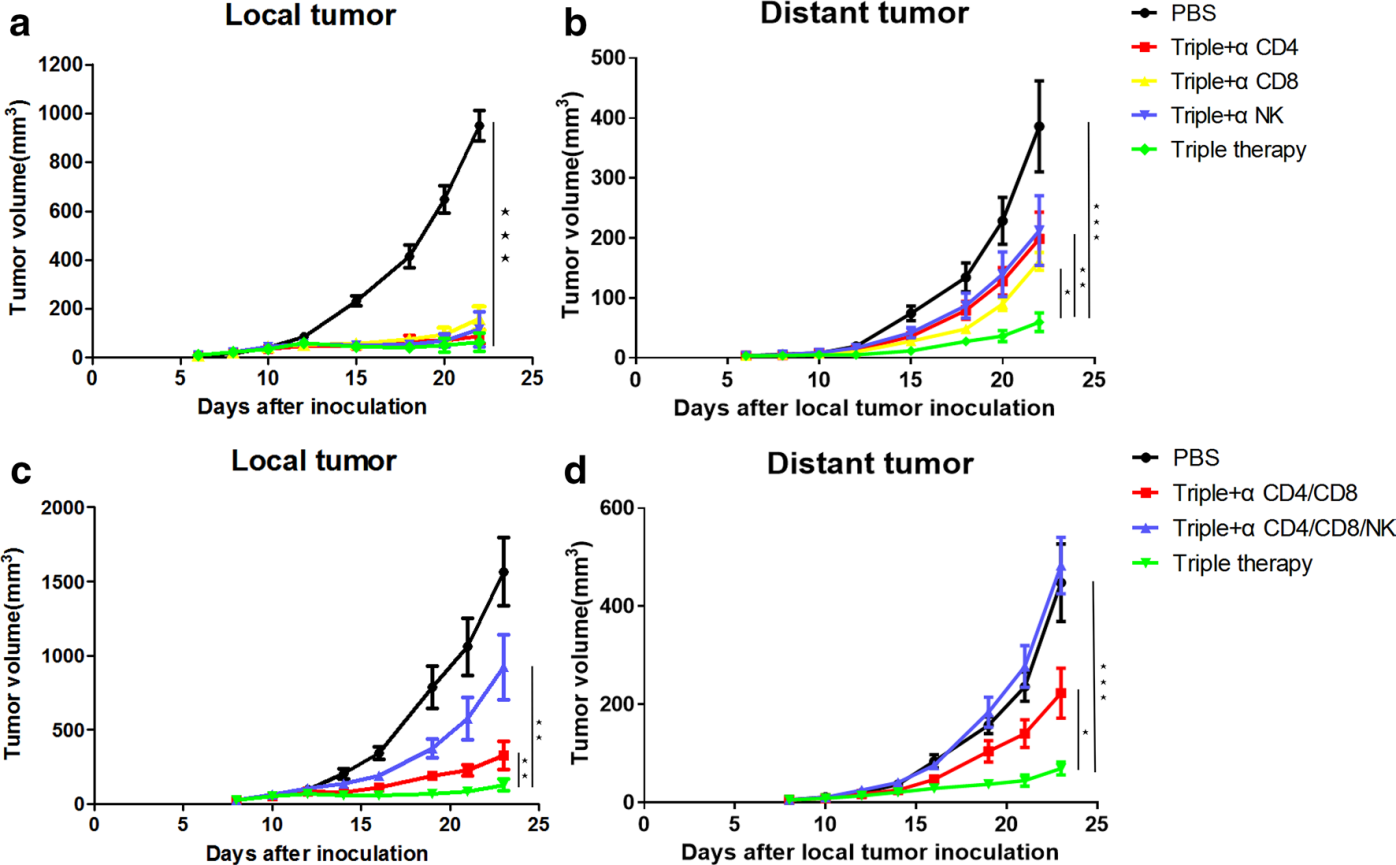
The antitumour effect of triple therapy depends on the full mobilization of adaptive immunity and innate immunity. TC1 tumour growth curves for the treated side (**a**) and untreated side (**b**) when CD4^+^ T cells, CD8^+^ T cells or NK cells were knocked out are shown. The TC1 tumour growth curves for the treated side (**c**) and untreated side (**d**) when both CD4^+^ T cells and CD8^+^ T cells or all CD4^+^ T cells, CD8^+^ T cells and NK cells were knocked out are shown

We conducted a cell depletion experiment to verify the importance of adaptive immunity and further explore the role of innate immunity in the effects of triple therapy. First, we used antibodies to knock out a certain cell type in mice, including CD4^+^ T cells, CD8^+^ T cells and NK cells (Supplementary Fig. 1). Regardless of what cell type was knocked out, triple therapy still exerted a strong antitumour effect on the treated side of the tumours, and no difference in the tumour volume was observed compared with the triple therapy group without antibody knockout (Fig. 5a). On the untreated side, although triple therapy still exerted a certain effect when CD4^+^ T cells, CD8^+^ T cells or NK cells were knocked out, the curative effect was significantly reduced (Fig. 5b). Thus, we speculated that in addition to adaptive immunity, innate immunity is also essential for mediating the effects of triple therapy. Next, we conducted a joint knockout experiment in mice. When we knocked out both CD4^+^ and CD8^+^ T cells, the efficacy of triple therapy was still strong; although the treated tumours tended to grow, the difference was not obvious. Triple therapy was still effective against untreated tumours (Fig. 5c). The systemic antitumour effect of the triple therapy disappeared only when CD4^+^, CD8^+^ T cells and NK cells were knocked out, and the distant tumour grew rapidly and even tended to surpass that in the control group (Fig. 5d). Thus, adaptive immunity and innate immunity play essential roles in antitumour immunity.

### Triple therapy systematically affects cytokine production in mice

**Fig. 6:**
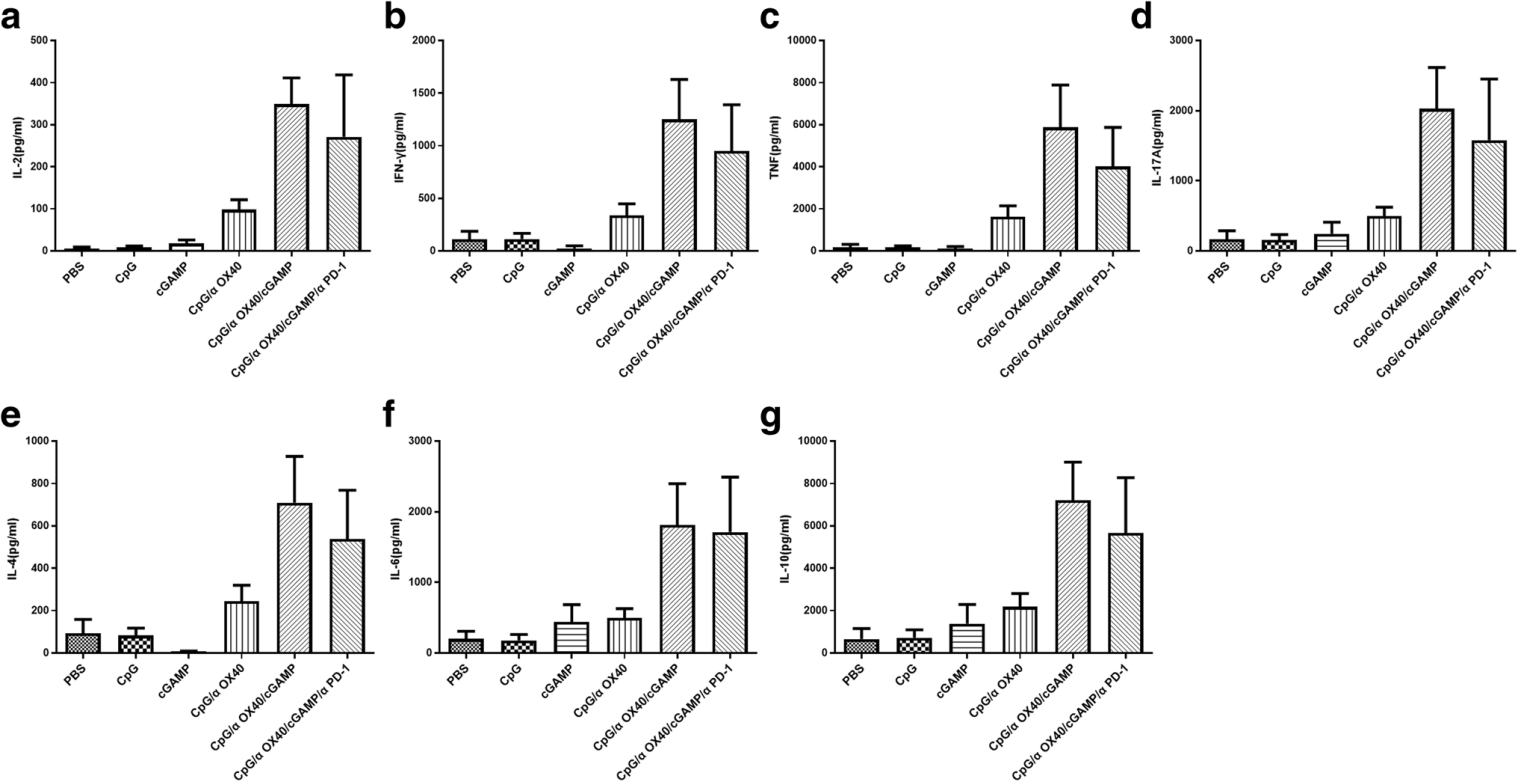
Triple therapy systematically increases cytokine production in mice. **a** IL-2, **b** IFN-γ, **c** TNF, **d** IL-17A, **e** IL-4, **f** IL-6 and **g** IL-10

In addition to immune cells, cytokines also play a crucial role in antitumour therapy. Thus, serum cytokine levels in mice after treatment were measured using CBA kits. After the different treatments, the levels of Th1 (IL-2, IFN-γ and TNF), Th2 (IL-4, IL-6 and IL-10) and Th17 (IL-17A) cytokines were noticeably increased, particularly in the triple therapy group, including both immunostimulatory cytokines and immunosuppressive cytokines (Fig. 6). After triple therapy, various cytokines are fully mobilized to exert a powerful antitumour effect.

### The combination of αPD-1 and triple therapy cannot enhance the systematic antitumour effect on αPD-1-resistant cell lines

**Fig. 7:**
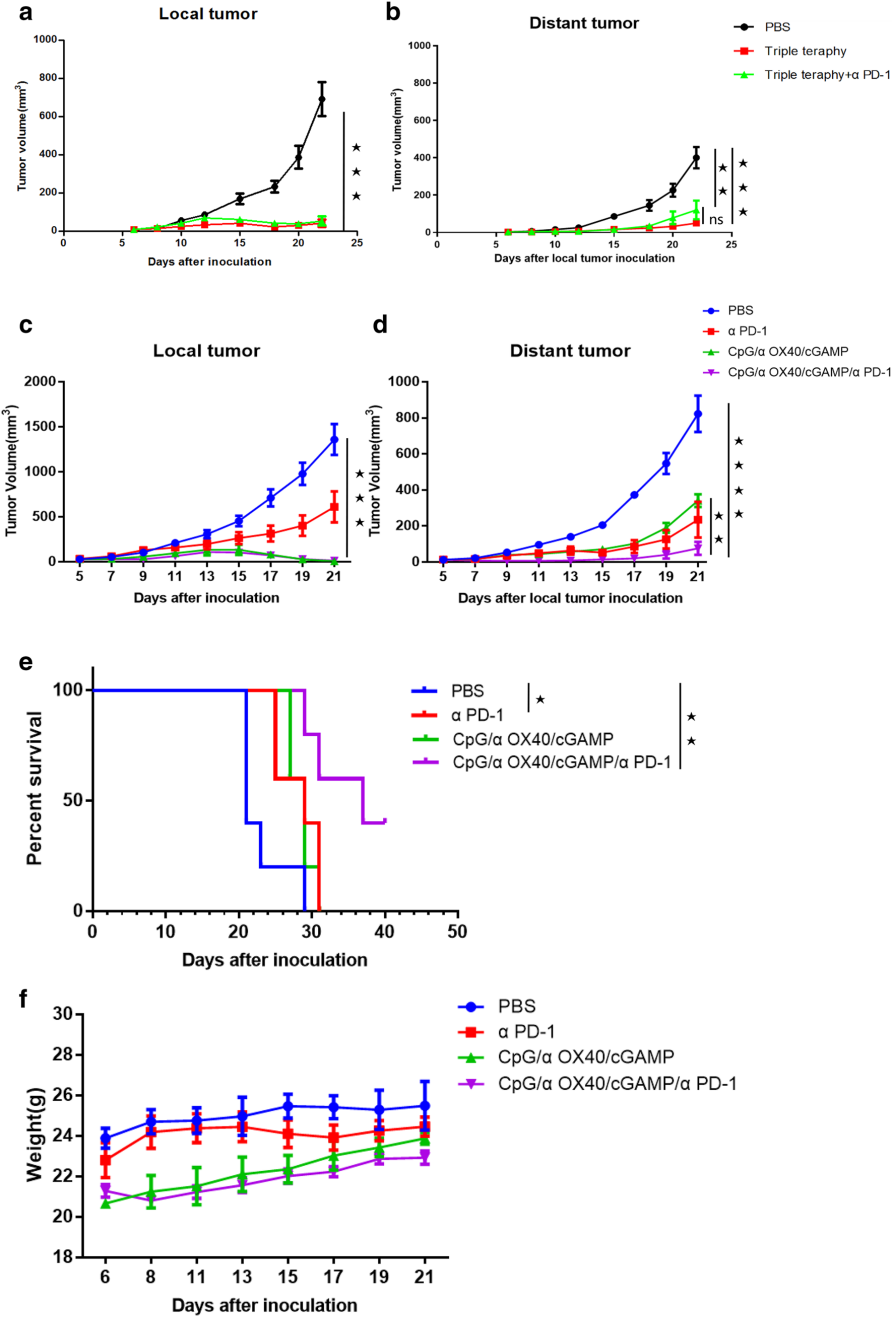
The addition of αPD-1 does not enhance the therapeutic efficacy of triple therapy in a TC1 mouse tumour model. The TC1 tumour growth curves for the treated side (**a**) and untreated side (**b**) are shown. In the PD-1-sensitive cell line CT26, aPD-1 and triple adjuvant had synergistic therapeutic effects. Tumour growth curves. Local tumour (**c**); distant tumour (**d**). **e** The survival of CT26 tumour-bearing mice. **f** Changes in the body weight of mice during the experiment

Finally, we wondered whether triple therapy combined with αPD-1 would exert a better effect, and thus, we conducted further animal experiments. Interestingly, when combined with αPD-1, the systematic antitumour effect of triple therapy was not better but tended to be worse (Fig. 7b), although it did not affect the antitumour efficacy on the treatment side (Fig. 7a). Coincidentally, the serum levels of cytokines were also consistent with this phenomenon, which may partially explain the findings. In this case, the application of the four drugs in combination did not increase the production of cytokines to improve the antitumour effect compared with triple therapy (Fig. 6). Furthermore, we explored combination therapy in the αPD-1-sensitive cell line CT26 and found that the systematic antitumour effect was better than triple therapy: distant tumours grew very slowly (Fig. 7c–d), and even the tumours of some mice subsided completely, achieving long-term survival (Fig. 7e and Supplementary Fig. 2). In terms of experimental safety, we observed the appearance and behavioural characteristics of the mice and recorded the changes in body weight of the mice during the experiment (Fig. 7f). None of the treated mice exhibited side effects such as weight loss, abnormal behaviour or accidental death.

## Discussion

In our study, we designed an effective treatment method for tumours that do not respond to PD-1 immune checkpoint inhibitors. By administering an in situ injection of three drugs in combination, namely the TLR9 agonist CpG, agonistic anti-OX40 antibody and STING agonist cGAMP, we not only significantly slowed the growth of tumours at the injection site but also altered the growth of distant tumours. This treatment even caused the regression of the tumours on the treatment side or on both sides in some mice, showing a strong systemic antitumour effect.

In situ injection of antitumour drugs is a practical strategy for cancer immunotherapy [21, 22]; in situ injection of CpG increases the expression of OX40 on CD4^+^ T cells in the tumour microenvironment, and the agonistic antibody OX40 triggers the immune response mediated by T cells and produces a systemic antitumour effect. Undeniably, CpG combined with in situ anti-OX40 antibody injection is a good drug combination [12]. However, this combination of drugs is not effective against all tumours. For example, in our study, the therapeutic effect of the in situ injection of CpG/αOX40 was not ideal in TC1 and B16 tumours, which are αPD-1-resistant malignancies. Surprisingly, when we combined these drugs with the STING agonist cGAMP, the antitumour effect was significantly enhanced and is expected to overcome the limitation of CpG/αOX40 therapy. Consistent with the results obtained with CpG/αOX40 therapy, CpG/αOX40/cGAMP therapy also fully mobilized CD3^+^ T cells. The flow cytometry results showed significantly increased proportions of CD4^+^ and CD8^+^ T cells in the spleen, and the infiltration of CD4^+^ T cells was dramatically increased in the distant tumours by approximately threefold compared with that in the control group. After the administration of triple therapy, immune cell infiltration into the distant tumour was dramatically increased, transforming the tumour from the original “cold” tumour to a “hot” tumour [23, 24]. Although CD8^+^ T cells play an important role in antitumour immunity, CD4^+^ T cells also play an indispensable role. In tumour immunity, CD4^+^ T cells activate CD8^+^ T cells through various mechanisms and promote their differentiation into cytotoxic T lymphocytes (CTLs) to maintain and strengthen the antitumour response of CTLs. On the other hand, even in the absence of CD8^+^ T cells, CD4^+^ T cells also directly kill tumour cells through an IFN-γ-dependent mechanism [25–28]. In terms of the therapeutic effect and the proportion of T cells, CpG/αOX40/cGAMP therapy is indeed better. Additionally, CpG/αOX40/cGAMP therapy fully mobilizes innate immunity. In the cell depletion experiment, the systemic antitumour effect of the triple therapy disappeared only when CD4^+^ T cells, CD8^+^ T cells and NK cells were all knocked out, indicating that both adaptive immunity and innate immunity played an essential role. Previous studies have found that an intratumoural injection of CpG/cGAMP also induces adaptive immunity and innate immunity, which further confirmed our findings [13].

In addition, triple therapy fully mobilized various cytokines, including Th1, Th2 and Th17 cytokines, which play important roles in regulating innate immunity and adaptive immunity. Th1 cytokines mainly include IL-2, IFN-γ, TNF and IL-12, which promote the activation and proliferation of cytotoxic T cells and dominate cellular immunity [29, 30]. Meanwhile, Th2 cytokines mainly include IL-4, IL-6 and IL-10, which play an important role in humoural immunity by regulating B cell activity and promoting antibody production [31–33]. Although IL-10 is an immunosuppressive cytokine, several studies have found that IL-10 induces effective antitumour immune surveillance and controls tumour growth [34–36]. In addition, IL-17 is mainly secreted by Th17 cells [37]. IL-17 plays different roles in the development of different tumours and may promote or inhibit tumour growth. Martin-Orozco et al. reported that IL-17 activates tumour-specific CD8^+^ T cells and inhibits the progression of B16-F10 melanoma with lung metastasis [38]. However, according to our current research results, it is difficult to infer the role of IL-10 and IL-17 because although the increase in IL-10 and IL-17 production was accompanied by the activation of antitumour immunity and the inhibition of tumour growth, a large number of other immunostimulatory cytokines were also increased. The activation of the OX40-OX40L signalling pathway has been reported to affect different T cell subsets, such as Th1, Th2 and Th17 cells [39–43]. According to Levy et al*.*, CpG/αOX40 therapy increases the expression of the cytokines IL-12, TNF and IFN [12]. Therefore, we speculate that the effect of the agonistic anti-OX40 antibody is fully activated after treatment with the combination of cGAMP with CpG/αOX40, which not only enhances the immune response mediated by Th1 cells but also promotes the production of Th2 and Th17 cytokines.

Finally, our study found that a new combination therapy also substantially suppressed the progression of tumours that failed to respond to PD-1 immune checkpoint inhibitors. Currently, two types of tumour immunotherapy are usually used: one is to relieve the inhibitory effect of cancer cells on immune cells by administering drugs, such as αPD-1/αPD-L1 and αCTLA-4 antibodies, an approach often compared to "releasing the brakes"[44, 45]; the other is to enhance the activity of immune cells to fight cancer by administering drugs, such as the anti-OX40 antibody and anti-41-BB antibody [46], which we often compare to "stepping on the accelerator"[47]. In our study, when the "removing the brakes" approach was ineffective against TC1/B16 cancer cells, we used the "step on the accelerator" approach. Excitingly, in situ injection of CpG/αOX40/cGAMP showed amazing systemic antitumour efficacy, promoting the secretion of a large number of cytokines and fully activating adaptive and innate immune responses. Furthermore, in αPD-1-resistant cell lines, when we combined αPD-1 with CpG/αOX40/cGAMP, the antitumour effect was not enhanced but appeared to be worse than that of CpG/αOX40/cGAMP alone. This result was also confirmed by the cytokine levels, which did not increase after the administration of the four drugs in combination compared with triple therapy. However, in the αPD-1-sensitive cell line CT26, we found a synergistic effect with this combination. Hence, we hypothesized that following the administration of triple therapy in αPD-1-resistant cell lines, tumour cells may escape the attack of immune cells through other escape mechanisms rather than by expressing the immunosuppressive molecule PD-1, such as the expression of other immunosuppressive molecules CTLA-4 [48] and TIM3 [49, 50], the increased expression of anti-apoptotic molecules [51, 52] or the decreased expression of antigens expressed by tumour cells [53, 54]. Moreover, the PD-1 immune checkpoint inhibitor may exert an opposite effect on shaping the environment created by triple therapy. We have not clearly elucidated the specific mechanism, and further in-depth studies are required.

Compared with other tumour immunotherapies, such as adaptive cell therapy, immune checkpoint inhibitors and tumour vaccines, the in situ injection of CpG/αOX40/cGAMP triggered an effective antitumour immune response while limiting the risk of systemic exposure and associated toxicity, which not only alters the tumour microenvironment at the injection site but also produces a systemic immune response and suppresses the development of distant tumours. Of course, our research also has some limitations. First, in situ injections are only achieved when the tumour is sufficiently large and accessible, which leads to the practical limitations of tumour treatment. Second, our analysis of the antitumour mechanism of triple therapy was not performed at a sufficient depth, and the reasons why αPD-1 does not enhance the curative effect of CpG/αOX40/cGAMP are not clear; therefore, the escape mechanism requires further exploration in future studies.

In summary, we designed an effective and safe drug combination. In situ CpG/αOX40/cGAMP injection generates a systemic antitumour immune response and simultaneously activates adaptive and innate immune responses, which is expected to overcome the bottleneck of ineffective treatment with PD-1/PD-L1 immune checkpoint inhibitors and provide benefits to certain cancer patients.

## Conclusion

In general, our study found that in situ vaccination with CpG/αOX40/cGAMP can be an effective treatment in a murine model of anti-PD1-resistant tumours. This method can not only activate both adaptive and innate immunity but also fully activate the production of cytokines, including Th1, Th2 and Th17 cytokines, exerting a non-negligible "heating" effect on the tumour immune microenvironment (Fig. 1). We believe that this kind of therapy can revolutionize the treatment of patients exhibiting αPD-1 resistance.

## Supplementary Information

Below is the link to the electronic supplementary material.

Supplementary file1 (PDF 68 KB)

Supplementary file2 (PDF 283 KB)

Supplementary file2 (DOCX 13 KB)

## Data Availability

The authors declare that the data supporting the findings of this study are available within the paper and its supplementary information files or available from the corresponding author upon reasonable request. Source data are provided with this paper.

## Abbreviations

CpG-ODN: Cytosine–phosphate–guanine oligodeoxynucleotides
cGAMP: Cyclic guanosine monophosphate–adenosine monophosphate
CTL: Cytotoxic T lymphocyte
FBS: Foetal bovine serum.
IDO: Indolamine-2,3-dioxygenase
iNHL: Non-Hodgkin’s lymphoma
MF: Mycosis fungoides
NSCLC: Non-small cell lung cancer
