# Supplementary figures and images for: Robust immune response stimulated by in situ injection of CpG/αOX40/cGAMP in αPD-1-resistant malignancy
Source: Cancer Immunol Immunother. 2021 Nov 3;71(7):1597–609. doi: 10.1007/s00262-021-03095-z (PMC9188536; doi:10.1007/s00262-021-03095-z)

**a**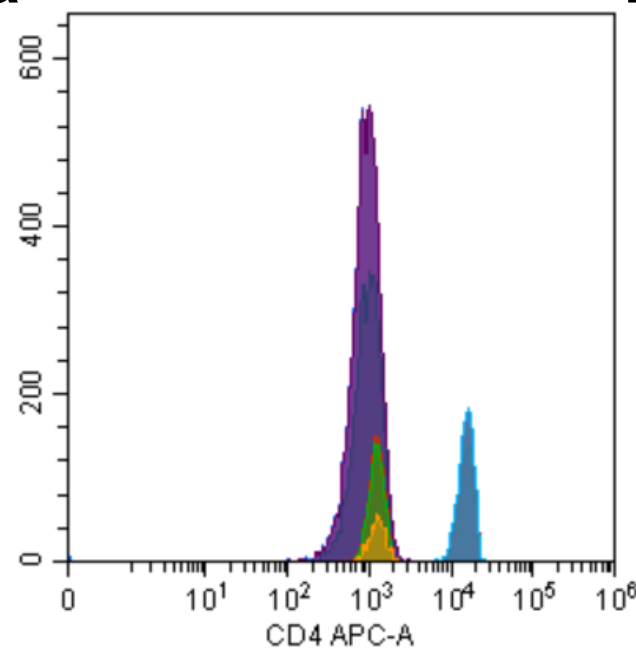**b**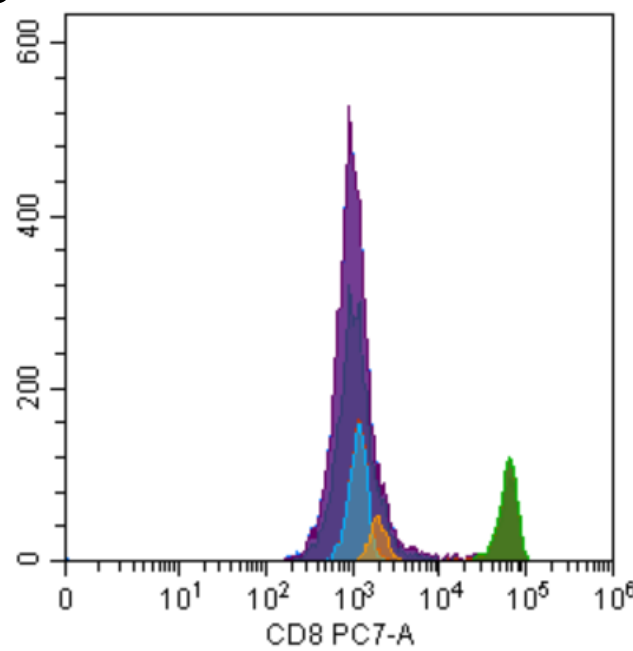**c**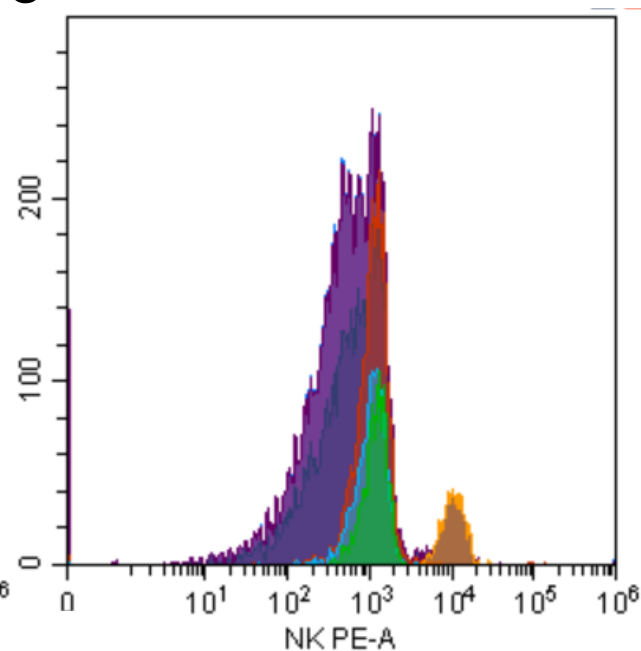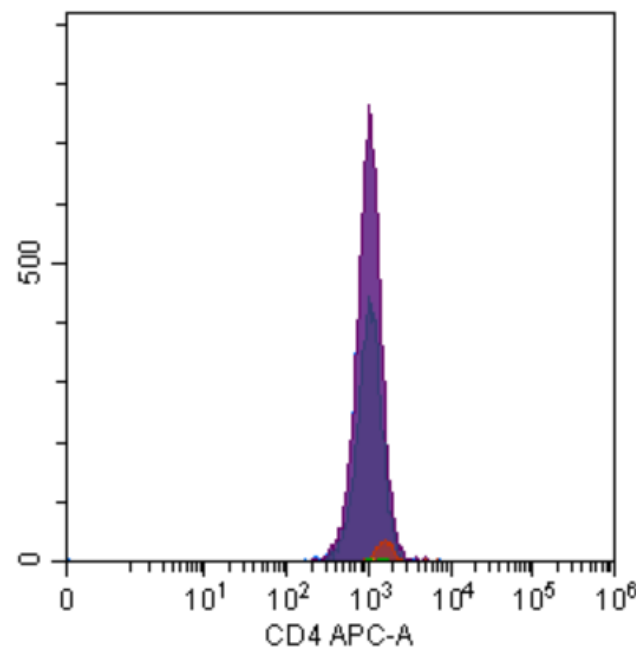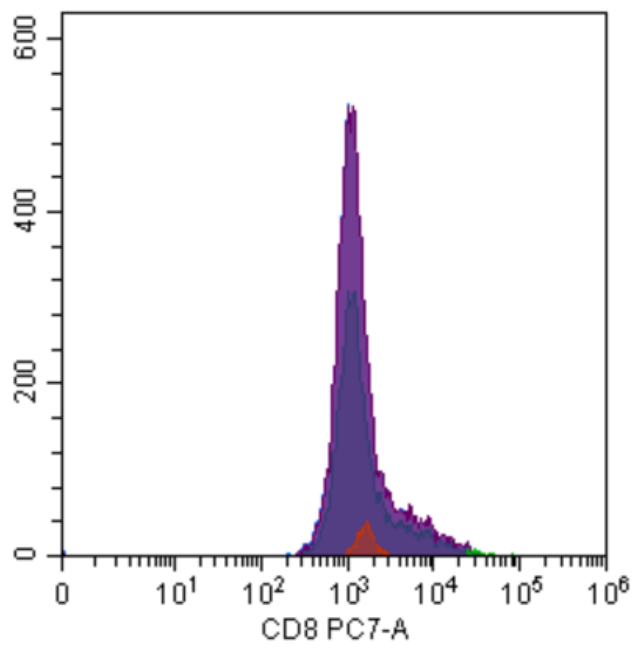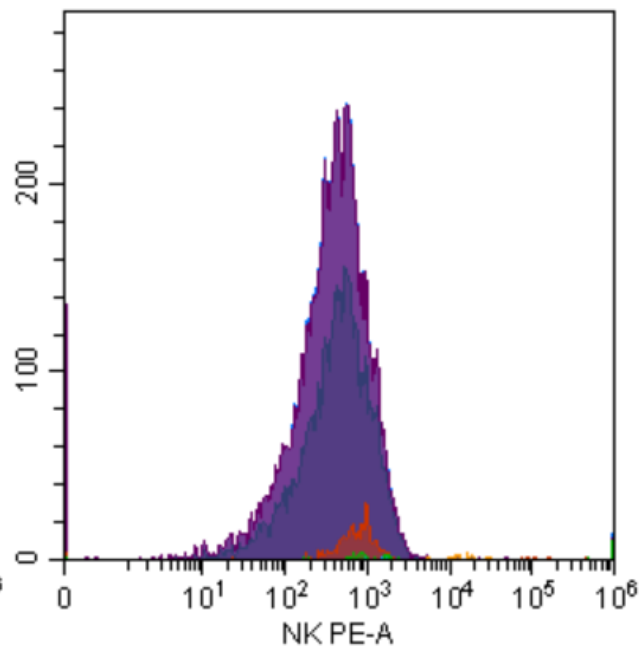

Supplement: Supplementary file 1 — Supplementary file1 (PDF 68 KB) [file 262_2021_3095_MOESM1_ESM.pdf]

PBS

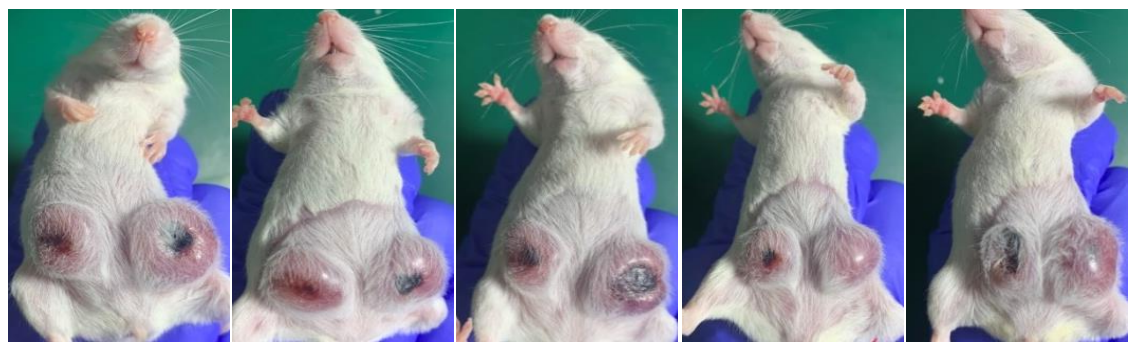

$\alpha$ PD-1

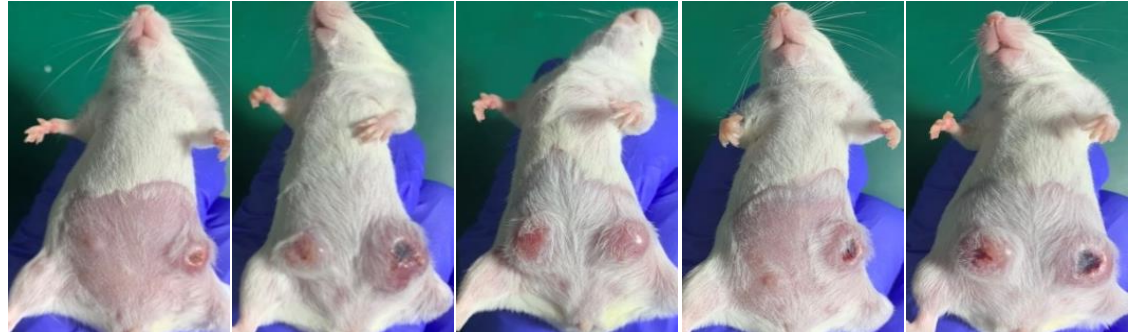

CpG/ $\alpha$  OX40/cGAMP

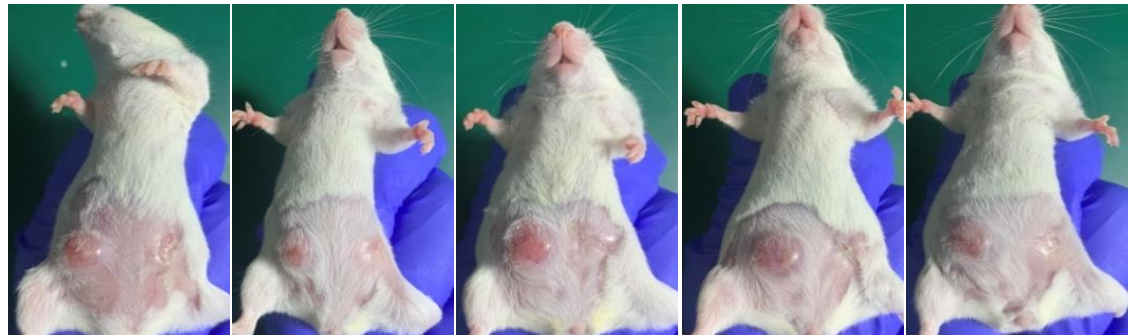

CpG/ $\alpha$  OX40/cGAMP/ $\alpha$  PD-1

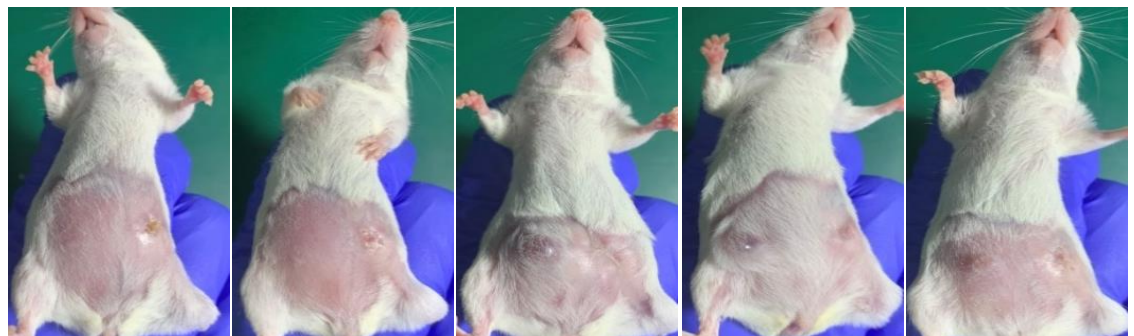

Supplement: Supplementary file 2 — Supplementary file2 (PDF 283 KB) [file 262_2021_3095_MOESM2_ESM.pdf]
