# Supplementary material for: Robust immune response stimulated by in situ injection of CpG/αOX40/cGAMP in αPD-1-resistant malignancy
Source: Cancer Immunol Immunother. 2021 Nov 3;71(7):1597–609. doi: 10.1007/s00262-021-03095-z (PMC9188536; doi:10.1007/s00262-021-03095-z)
Supplement: Supplementary file 3 — Supplementary file2 (DOCX 13 KB) [file 262_2021_3095_MOESM3_ESM.docx]

Supplementary Figure 1. The depletion states of CD4^+^ T cells (a), CD8^+^ T cells (b) and NK cells (c) were validated by performing flow cytometry analysis of blood samples and showed that the cells were basically removed.

Supplementary Figure 2. Images of CT26 tumours in mice on day 21 after tumour cells were implanted subcutaneously
